# Screening of nucleotide variations in genomic sequences encoding charged protein regions in the human genome

**DOI:** 10.1186/s12864-017-4000-3

**Published:** 2017-08-08

**Authors:** Sabrine Belmabrouk, Najla Kharrat, Rania Abdelhedi, Amine Ben Ayed, Riadh Benmarzoug, Ahmed Rebai

**Affiliations:** Centre de Biotechnologie de Sfax, Laboratoire de Procédés de Criblage Moléculaire et Cellulaire, PoBox ‘1177’, 3018 Sfax, Tunisia

**Keywords:** Charge cluster, Genetic variant, Mutation, Substitution, Deletion, Insertion

## Abstract

**Background:**

Studying genetic variation distribution in proteins containing charged regions, called charge clusters (CCs), is of great interest to unravel their functional role. Charge clusters are 20 to 75 residue segments with high net positive charge, high net negative charge, or high total charge relative to the overall charge composition of the protein. We previously developed a bioinformatics tool (FCCP) to detect charge clusters in proteomes and scanned the human proteome for the occurrence of CCs. In this paper we investigate the genetic variations in the human proteins harbouring CCs.

**Results:**

We studied the coding regions of 317 positively charged clusters and 1020 negatively charged ones previously detected in human proteins. Results revealed that coding parts of CCs are richer in sequence variants than their corresponding genes, full mRNAs, and exonic + intronic sequences and that these variants are predominately rare (Minor allele frequency < 0.005). Furthermore, variants occurring in the coding parts of positively charged regions of proteins are more often pathogenic than those occurring in negatively charged ones. Classification of variants according to their types showed that substitution is the major type followed by Indels (Insertions-deletions). Concerning substitutions, it was found that within clusters of both charges, the charged amino acids were the greatest loser groups whereas polar residues were the greatest gainers.

**Conclusions:**

Our findings highlight the prominent features of the human charged regions from the DNA up to the protein sequence which might provide potential clues to improve the current understanding of those charged regions and their implication in the emergence of diseases.

**Electronic supplementary material:**

The online version of this article (doi:10.1186/s12864-017-4000-3) contains supplementary material, which is available to authorized users.

## Background

Charge clusters (hereafter abbreviated as CCs) are protein regions characterized by a high concentration of charged residues (Aspartic and Glutamic acids: negative; Lysine and Arginine: positive). Karlin [1] defined and identified CCs in proteins as 20 to 75 residue segments with high net positive charge (Positive Charge Clusters, PCCs), high net negative charge (Negative Charge Clusters, NCCs), or high total charge (mixed charge clusters) relative to the overall charge composition of the protein. Until 1995, Samuel Karlin and co-workers [2] have largely studied CCs in different species. However, since then, there has been no comprehensive and exhaustive study of the occurrence of CCs in proteomes. For this purpose, we recently developed the FCCP («Finding Charge Clusters in Protein sequences») program [3] to detect clusters of residues of the same charge possibly interrupted by uncharged residues but free of any residue of the opposite charge. We used FCCP to study the proteome wide occurrence of CCs and their distribution in human proteins. The program has detected 526 PCCs and 1628 NCCs with an average size of 24.2 ± 3.9 amino acid (aa) for NCCs and 27.8 ± 7.6 aa for PCCs. Furthermore, we found that 1.6% and 3.4% of human proteins contain positive and negative charge clusters, respectively. Interestingly, NCCs are three times more prevalent than positive ones. The PCCs were found to be more frequently localized into the functional domains of the human proteins. In contrast, NCCs are most of the time present in C-terminal domains. Moreover, according to the Gene Ontology classification, proteins with CCs are mostly involved in binding functions. In other words, proteins having CCs are mainly binders of nucleic acids and other proteins. Additionally, CCs are also abundant in domains of transcriptional factors, catalytic proteins, transporter proteins, regulatory proteins and signalisation paths [3]. For instance, Aifa et al. [4] proposed a general model of intracellular receptor dimerization of the EGFR family based on the interaction of a positive 13 amino acid peptide (P13+) with a negative 13 amino acid peptide (P13-). Moreover, in a recent unpublished study, we found out that CCs are structurally mainly intrinsically disordered or contained in intrinsically disordered proteins. This result was also reported by Choura and Rebai [5].

Due to their electrostatic potential, CCs are capable to bind other macromolecules and to help complex formation. Firstly, CCs of opposite charges in two different proteins help to multimeric complex formation [4, 6]. Secondly, Sheinerman et al. [7] reported that CCs localized at the interface of protein-protein complex could enhance the stability of the complex. On the other hand, electrostatic interactions around “charge centres” in protein structures were shown to play a key role in the folding and stability of the protein architecture [8].

Disrupting the net charge of these structurally critical regions could destabilize the protein and affect its function [9]. Hence, in most reported studies, mutations in charged residues result in complex destabilization. For example, the substitution of a charged residue located at the interface of protein-protein complex by a variant decreases the stability of the complex; the removal of a charged residue that forms a salt bridge across the interface in the complex leaves the charged partner without favourable pairwise interactions. Vreken et al. [10] reported that changes in the buried charge on the interface of proteins or near to a ligand biding site could lead to the onset of diseases. In general, all mutations of a charged residue to another charged residue were found to be unfavourable events [11].

Genetic variations such as Single Nucleotide Polymorphisms (SNPs), frame shifting deletions and insertions and nonsense mutations may have a large effects on protein functions and therefore are likely to be disease-causing events. Non synonymous variants can affect the stability of proteins and protein-protein complexes [11] as well as protein folding, interaction sites, protein solubility and stability [12]. Therefore, they can alter the function of the protein by changing the stability of its native structure and/or its binding properties. The mechanisms of the effect of non synonymous variants on the stability of the protein are very variable: geometric constraints, physicochemical effects, inversion of a charge in a salt bridge, or the disruption of hydrogen bonds [13]. For instance, among the most frequent effects of non synonymous SNPs on protein stability is the loss of hydrogen bonds (21%, 1/4 of which involves a charged group) and buried charged residues (14%), where the variant introduces an isolated charge cancelling electrostatic accessibility [14].

Very few studies have tried to unravel the riddle of these highly charged regions (CCs) seen in human proteome. The objective of this work is to elucidate the variations occurring in the corresponding genomic sequences of charged regions in human proteins using the dataset of CCs that we previously detected by the FCCP program [3]. The outline of the paper is as follows; we first describe the dataset, then we present the distribution analysis of genomic variants in these CCs genes, full mRNAs and proteins, as well as their clinical significance, molecular consequences, types and classification according to the Minor Allele Frequency (MAF). Finally, an overall analysis of non synonymous and synonymous variants effect on the amino-acid sequences of CCs and the protein in general was evaluated.

## Methods

### Dataset

The human CCs dataset was retrieved from our CCs databank detected by the FCCP program [3]. It is a local databank containing a set of 526 and 1628 human PCCs and NCCs sequences found in 498 and 1435 proteins, respectively. The dataset details are given in Additional file 1.

### Data processing

Our study was conducted mainly with a set of scripts written and executed in R language (Additional file 2). We proceeded in two steps (Additional file 3):

**Step 1:** Firstly, the protein existence was manually checked in Uniprot [15] database. Then, gene identifier corresponding to each screened protein was retrieved from Uniprot [15]. Nonetheless, the count of starting proteins has been reduced for several reasons: whether proteins were obsolete, or having different sizes than available in our CCs local databank or substituted with other proteins or having changed their names.

In order to solve these problems cited above, we proceeded according to the following steps:

- *Proteins with changed names*: we checked their existence in the initial list of proteins, otherwise, we screened again the new protein by the FCCP program to confirm the presence of CCs.
- *Proteins with changed sizes*: the new and the old proteins were aligned using BLASTp [16] in order to verify the position of the CC and then screened by FCCP to confirm the existence of the CC at the same position.

**Step 2:** Later, we searched GenBank [17] with the gene identifier of the corresponding screened protein so as to localise the CC and its protein in the genomic level (in the gene). In case that the protein size in the «Gene Table» does not match that of the starting protein (the FCCP output), we Blast aligned both proteins in order to verify that it is indeed the same protein and in some cases, recalculating the exact position of the cluster was required. Furthermore, all proteins with no gene entry or data in GenBank [17], were removed from the study. Then, the position of the CC was searched in the gene. Using an R script (Additional file 2), the data of each CC was kept in a data frame which contains the gene identifier, the number of corresponding chromosome, the Uniprot identifier of the protein, the start and final positions of the gene, the protein, the full mRNA and the CC in the chromosome.

The final data frame, where all CCs and their corresponding data were listed, was used to recover variant data in the gene, the full mRNA, the CC (exonic sequence) and in the CC with its intronic sequence (when CC is encoded by more than one exon, exon + intron). Finally, we kept 1020 NCCs and 317 PCCs. In order to avoid possible discrepancies between RefSeq [18] and Uniprot [15], only 768 NCCs and 50 PCCs have been considered in the assessing of amino-acids variations.

### The recovery of the variant data

In order to recover the variant data, the Variation Viewer [19] was searched with the gene positions. The retrieved variant list was treated with an R script (Additional file 2) for subsequent manipulation. Finally, a database of variants in genes, full mRNAs, exonic and exonic + intronic sequences of CCs was generated. The final result is a data frame containing the molecular consequences, the clinical significances, the types, the classification according to 1000 Genomes, GO-ESP and Exome-AC MAFs of variants in each CC.

So as to compare the variant densities between the gene, the full mRNA and the CC (exonic and exonic + intronic sequences), the variant fraction was calculated by dividing the count of the variants by the sequence size:

Variant Fraction = $$ \frac{Variant count in the genomic sequence}{sequence size\ \left( nucleotide count\right)} $$

The genomic sequence might be that of the CC, the gene, the full mRNA, the exon, the intron or exon + intron.

### Analysis of amino acid variation occurring within CC

In this part of the study, the «Protein change» datasets of the «Gene table» of 768 NCCs and 50 PCCs were searched for substitutions, Indels and synonymous variants (among substitution) affecting both charged and uncharged residues which were classified into four groups according to the physicochemical properties of their side chains: hydrophobic (Ala, Val, Leu, Ile, Met, Phe, Trp, Pro), polar (Gly, Ser, Thr, Cys, Tyr, Asn, Gln, His), acidic (Glu and Asp) and basic (Lys and Arg). The amino acids variations were segregated according to the change of their physicochemical properties (polar, negative, positive and hydrophobic). The differences between distributions of types of variants according to the amino acid groups were assessed. Also exchanges between groups of amino acids were evaluated. This was achieved using an R script (Additional file 2).

### Databanks and tools

In our work, local and online databanks and bioinformatics tools were used. Variation Viewer [19] (release 1.5) was used as a tool for navigating variant data in NCBI’s databases: dbSNP (SNP and multiple small-scale variations database, release 149), dbVar (Genomic structural variation database, release December 2015) and ClinVar (Data base for genomic variation and its relationship to human health, release January 2016). Thus the Variation Viewer helped us to classify our variants according to their clinical significances (pathogenic, probably pathogenic, risk factor, likely benign, benign...), types (single nucleotide variation, deletion, insertion..), molecular consequences (missense, nonsense, synonymous, inframe...) and MAF (Minor Allele Frequency).

Classification of variants according to their Minor Allele Frequencies (MAFs) was conducted using three sources: 1000 Genomes project (release 20,110,521), GO Exome Sequencing Project (GO-ESP, release September 6, 2011) and the Exome aggregation Consortium (EX-AC, release 0.3.1). The MAF’s classification was carried out according to three intervals: < 0.005, [0.005, 0.01], [0.01, 0.05] and ≥0.05.

Finding Charge Clusters in Protein sequences (FCCP), the tool used for detecting significant and disjoint CCs [3] in the human proteome (available details in the introduction section), implements an algorithm based on the original method of score [20].

### Statistical tests

The chi-square test (χ^2^) was used to determine whether the distribution of variant types or the molecular and clinical consequences of variants or the classification according to the MAF (based on 1000 Genomes, EX-AC and GO-ESP) was significantly different between NCCs and PCCs or not. It was also used in order to assess the significance of differences between distribution of variant types (substitution, insertion, deletion, synonymous) according to the amino acid groups (hydrophobic, polar, basic and acid). The ANOVA (Analysis of Variance) was used to assess the significance of the differences in variant frequencies between the gene, the full mRNA, the exonic and exonic + intronic sequences of CC. The multiple mean comparison tests (Tukey HSD) was used in order to determine the specific differences between the above mentioned groups.

## Results

### Distribution of CCs within the human genes

The encoding sequence of a CC could be entirely included in one exon or spread over several exons and consequently it may be interrupted by introns. We found that the distributions of NCCs and PCCs in human genes are similar. In fact, in both cases, 67% of them are encoded by an unique exon, while 30% to 31% are encoded by two exons (separated by an intron) and only 2% to 3% are encoded by three or more exons (Additional files 4 and 5).

### Variant distributions

**Fig. 1:**
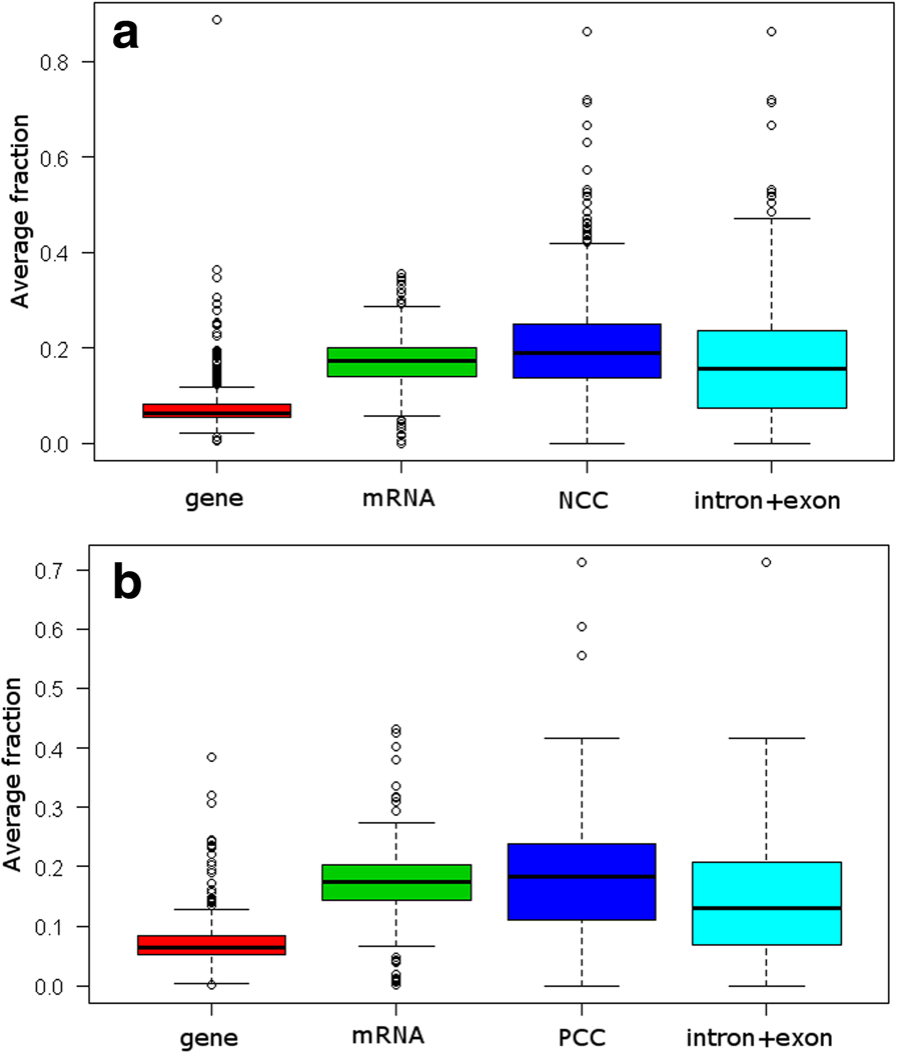
Average fraction of variants within charge clusters and their corresponding background sequences. The variant fractions were calculated according to the formula in the Methods section (The recovery of the variant data) within negative (**a**) and positive (**b**) charge clusters and their corresponding genes, full mRNAs and intron + exon sequences. Variant fractions within clusters of both charges (**a** and **b**) are in average bigger than those within their genes, full mRNAs and exon + intron sequences

**Fig. 2:**
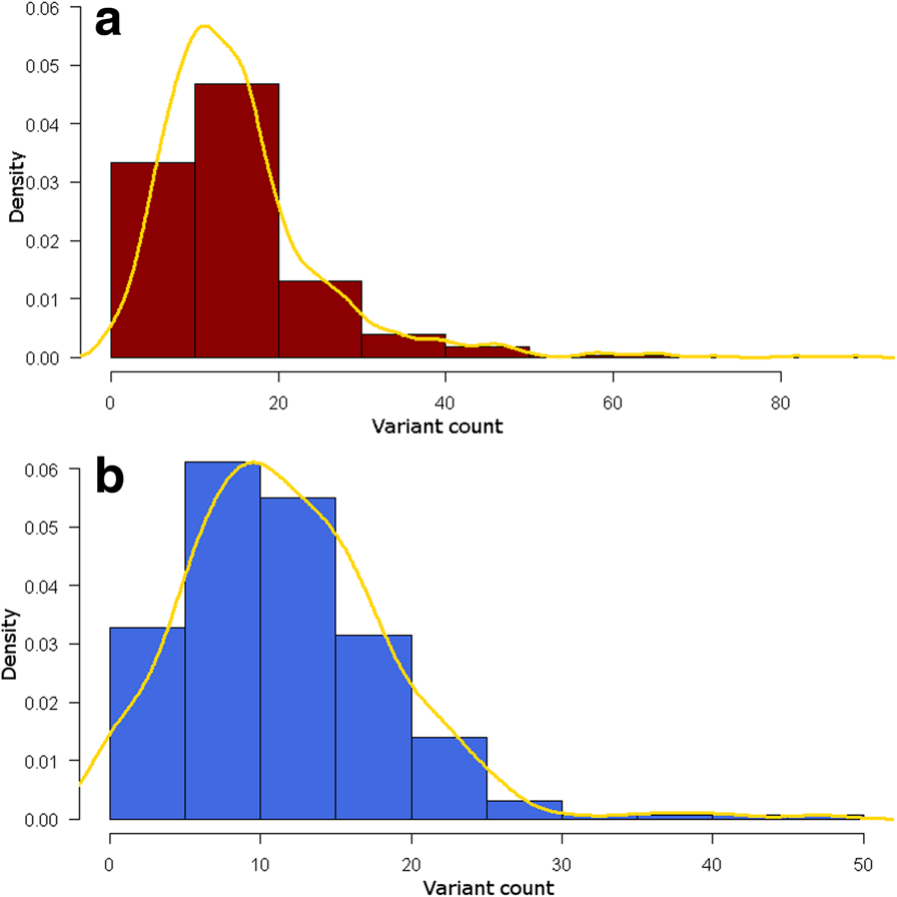
Distributions of variant counts within negative (**a**) and positive (**b**) charge clusters. The density of frequencies are represented according to the variant count in NCCs (**a**) and PCCs (**b**)

The CCs encoding sequences were found to be significantly more exposed to variants than their corresponding genes, full mRNAs and exonic + intronic sequences (*p* ≥ 10^−6^, Fig. 1). Furthermore, within NCCs the variant average count is 15.4 ± 10.2, which represents one variant every 4 bp; whereas it is 11.8 ± 6.9 within PCCs which represents about one variant every 5 bp. These findings suggest that the CCs are considered as variant rich regions (Fig. 2). Besides, it was found that the exonic sequences of NCCs are richer in variants than those of PCCs (*p* = 0.0036). This could be due to the fact that NCCs are in average longer than PCCs [3].

### Classification of variants detected in CCs of the human genome

Only variants detected in the exonic sequences of CCs and their corresponding full mRNAs were considered for further investigations; their types, clinical and molecular consequences and MAFs (according to 1000 Genomes, Go-ESP and EX-AC) classifications were conducted using the Variation Viewer data.

#### Classification according to the clinical signification

**Fig. 3:**
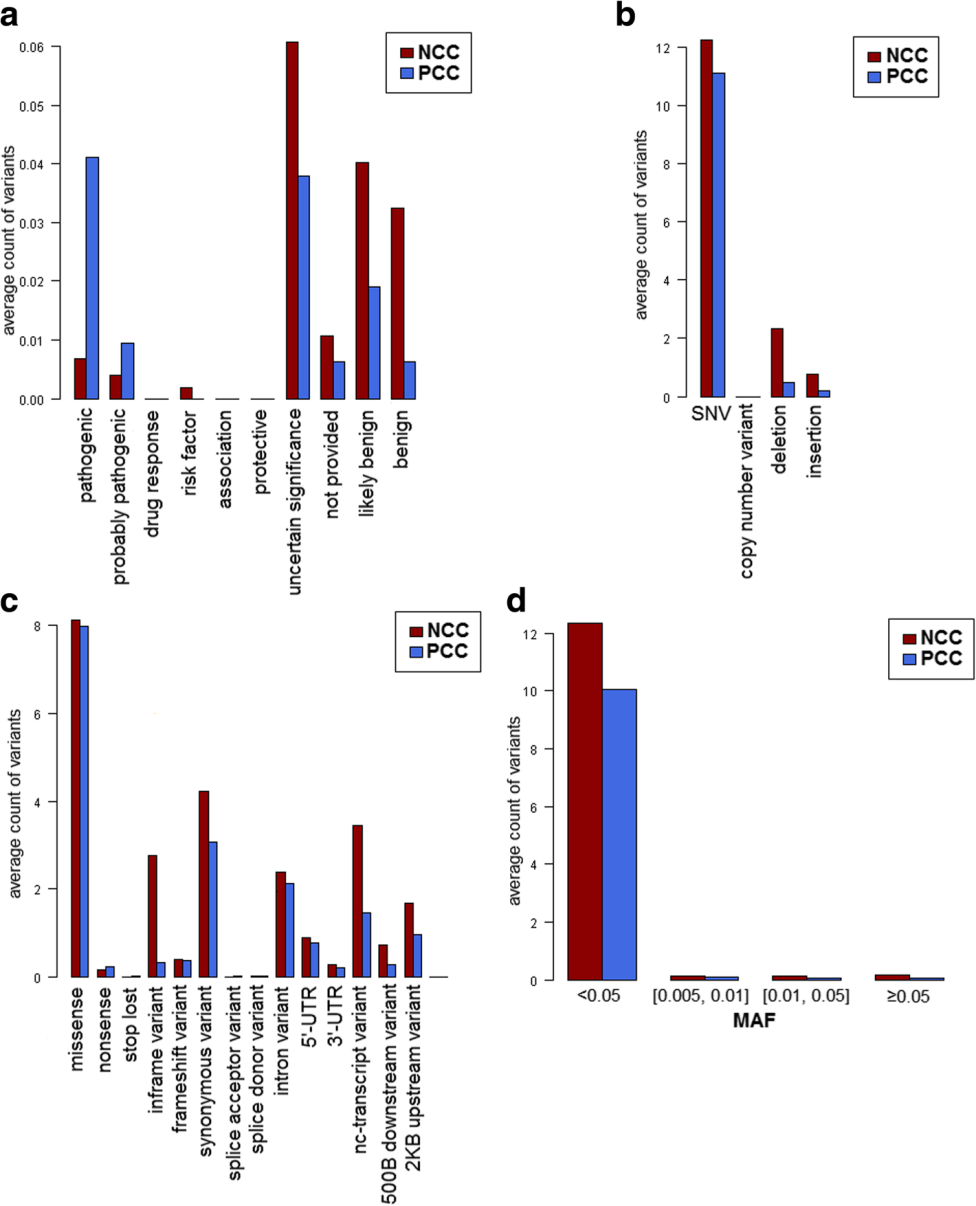
Distribution of variants detected within CCs according to the Variation Viewer data. The average count of variants are represented according to their clinical significances (**a**), types (**b**), molecular consequences (**c**) and minor allele frequencies (ExAC-MAF) (**d**) for negative charge clusters (NCC, red) and positive charge clusters (PCC, blue). The classification of variants was provided by Variation Viewer (Details in Materials and Methods section). The plot shows that PCCs are richer in pathogenic and probably pathogenic variants than NCCs (*p* = 2.65 × 10^−7^), which are richer in benign and likely benign variants (*p* = 0.0432). Moreover, the majority of variants are single nucleotide variants (SNV, 80%), missense (*p* = 2.2 × 10^−16^) and rare (MAF < 0.005; *p* = 5 × 10^−5^)

Interestingly, variants within exonic sequences of PCCs were found to be mainly pathogenic (34% from specified variants, *p* = 2.65 × 10^−7^). Moreover, these variants are similarly frequent in the exonic sequences of PCCs as in their corresponding full mRNAs. Conversely, variants within exonic sequences of NCCs were more likely to be benign or likely benign (46% from specified variants, *p* = 0.0432). In contrast to PCCs, benign and likely benign variants are more frequent in the exonic sequences of NCCs than in their corresponding full mRNAs (Fig. 3a).We should mention that the not specified variant category was discarded.

In summary, NCCs are subject to much less sequence variation that are pathogenic than their background sequences whereas PCCs have the same probability of being pathogenically mutated than the background sequences. Accordingly, we might conclude that NCCs tend to be more conserved than PCCs indicating that a variation in an NCC is rarely pathogenic and most of them are synonymous or conservative.

#### Classification according to the variant type

The main variant types in both full mRNAs and exonic sequences of CCs are: «Single Nucleotide Variant» (SNV), which represents 80% of variants found in CCs, «Deletions» and «Insertions» regardless of the charge of the cluster (Fig. 3b). Nevertheless, NCCs are significantly richer in these three variant types cited above than the PCCs (*p* = 1.4 × 10^−16^) in both full mRNAs and exonic sequences. This latter result may be due to the fact that NCCs are significantly longer than PCCs [3].

#### Classification according to the molecular consequence

The direct impact of a variant is manifested by a change in the nucleotide sequence. We talk about the molecular consequence of the variant, which may be a splicing variation leading, for example, to a modified 3′ region of an intron or a stop lost or a nonsense variant. Whatever is the charge of the cluster, the «missense» variants are the most important representing 33% and 44% in NCCs and PCCs respectively (*p* = 2.2 × 10^−16^; Fig. 3c). They are twice as numerous as the «synonymous» variants that came in the second place. There is on average 8 missense variants by cluster (of both charges).

#### Classification according to the minor allele frequency (MAF)

Interestingly, most variants found within CCs are rare (MAF < 0.005), according to the classification of 1000 Genomes, GO-ESP and EX-AC (*p* = 2 × 10^−4^, *p* = 8.4 × 10^−5^ *, p* = 5 × 10^–^^5^ respectively, Fig. 3d); in fact, variants with a MAF < 0.005 are the most prevalent class for both types of CCs (85% and 78% in PCC and NCC respectively, according to ExAC MAF). Note that, the non specified variants (no data available on allele frequency) were eliminated in calculation.

### Assessing the amino acid variations in the human CCs

Probably the most important question is to know whether a trend exists for the genetic variations that occur in charged regions of proteins, i.e. do variations follow some pattern according to the groups of amino acids or are they randomly distributed. To answer these questions, we evaluated the occurrence of four variant types: substitution, deletion, insertion and synonymous according to groups of amino acids (polar, hydrophobic, basic and acidic).

#### Within NCCs

**Fig. 4:**
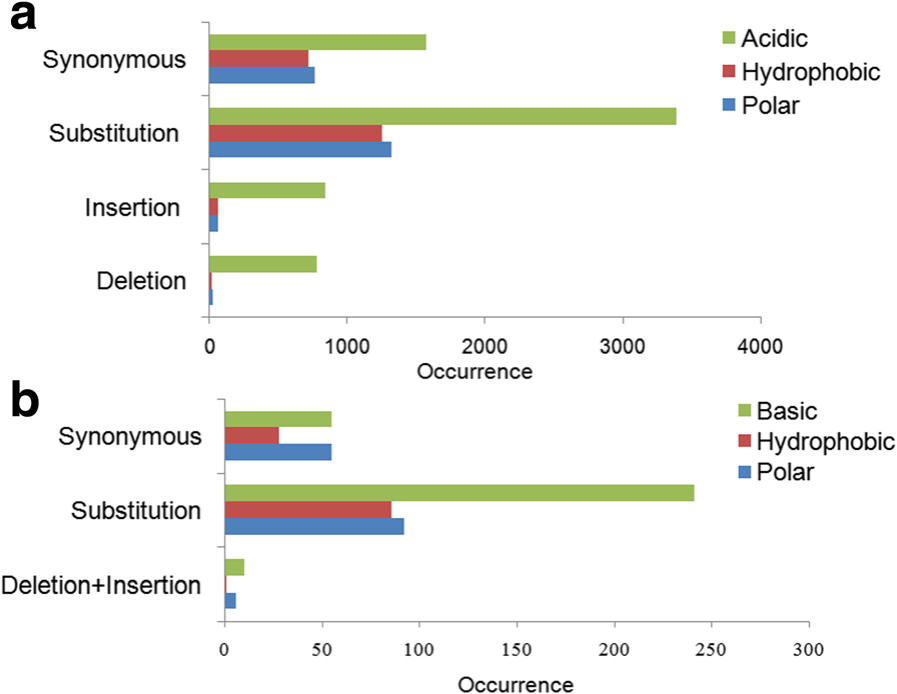
Distribution of variant type according to the amino-acid group. The variant types (synonymous, substitution, insertion and deletion) are represented according to the amino-acid groups (acidic, hydrophobic and polar) for negatively charged clusters (**a**) and (basic, hydrophobic and polar) for positively charged clusters (**b**)

**Fig. 5:**
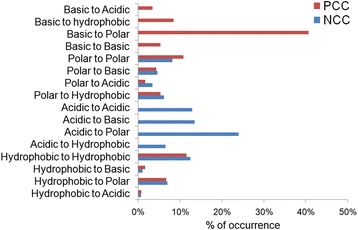
Percentages of the occurrence of substitutions between and within groups of amino acids. Blue: in negative charge clusters. Red: in positive charge clusters. Substitutions of charged residues by polar ones are the most frequent

Results revealed that there is a significant difference between the occurrence of the four variation types according to groups of amino acids (*p* = 1.28 × 10^−179^). Obviously, the acidic amino acids are the most affected by the variations, since they are the most frequent. Actually, Glutamic and Aspartic acids constitute 34.5% and 22%, respectively, of the overall count of mutated amino-acids. At the same time, both polar and hydrophobic residues are equally mutated. In addition, the substitution is the most common variation type (55%) followed by the synonymous variants (28%) than both deletion and insertion (16% together) (Fig. 4a). As a matter of fact, there are 5965 substitutions that occur within the NCCs, among which, 57% affected Aspartic and Glutamic acids. Substitutions of negative by polar residues are the most common (24%) followed by substitutions from negative to negative and negative to positive, that were equally prevalent. However, only 11.5% of negative residues were found to be substituted by hydrophobic residues (Fig. 5).

Furthermore, we found that 469 insertions occur within the NCC dataset and the number of the inserted amino acids ranged from one (54% of cases) to twelve. Of course, the occurrence of insertions was found to decrease according to the number of inserted residues. Surprisingly, the acidic residues are the most inserted; they constitute between 50 and 93% of each insertion category (i.e. number of inserted amino acids). For instance, in the one-amino-acid insertion category, the number of inserted acidic amino acids is 234 out of 252 whereas only 4 hydrophobic and 10 polar residues out of 252 were found to be inserted. Also, only 8 basic amino acids were found to be inserted in all NCCs (Additional file 6).

Similarly, the acidic amino acids are the most deleted (53%). They are also the most affected by synonymous variants (51%). This is expected since NCCs are characterized by their richness in Glu and Asp (Additional file 7).

#### Within PCCs

Comparatively to the NCCs, there is a significant difference between the occurrence of the four variation types according to the group of residues (*p* = 3.3 × 10^−4^). As expected, the basic amino acids are the most affected by the variation (*p* = 0.0013), since they are the most frequent residues. The substitution is the most common variation type (96%) followed by the synonymous variants than both deletion and insertion which are infrequent (Fig. 4.B). In fact, there are 419 substitutions that occur within PCCs, among which 57% affected Lys and Arg. Basic residues are most frequently substituted by polar ones (41%) then by hydrophobic (8%) and positive and negative residues (5% and 3% respectively). In summary, basic residues are substituted in 85.5% of cases by polar ones including charged ones (positive and negative) (Fig. 5).

Basic residues were found to be also the most deleted (8 out of 21) followed by the polar then the hydrophobic residues. Unlike the NCCs, insertions within PCCs are very rare; only 2 positive and 3 polar residues were inserted in the set of the PCCs. Both polar and basic residues are the most affected by the synonymous variants. However Arg is the most mutated (32%), but surprisingly, the second most mutated residue is Pro (Additional file 8).

## Discussion

As was mentioned in the introduction section, charged regions in human proteome were not granted the same interest as other types of biased regions, e.g. the hydrophobic regions. The importance and urgency of continued research on charged regions (CCs) is underlined by their range of important interactions and functions [3, 21–23], including their involvement in human diseases [24, 25].

In this respect, variations in encoding sequences of CCs may lead to such diseases. In fact, based on results of classification according to the MAF, variants occurring within CCs were found to be mostly rare (MAF < 0.005). According to Tennessen et al. [26], this shows that the encoding variants of charged regions are population-specific and potentially deleterious. Actually, many researchers reported that residue substitutions observed rarely are likely to be radical; they are rare because functional changes in specialized proteins are most often deleterious and are purified from the population by natural selection [27–31]. Besides, the molecular consequences of the variants found in the exonic sequences of CCs showed a dominance of the missense variants that cause a change in the amino acid sequence which may lead, to a non-functional protein and therefore to disease emergence.

For instance, two mutations (c.3879dupA; Glu1294Argfs) [32] and (c.3070C > T; Arg1024Ter) [33] located in the C-terminal transactivation domain of the Histone acetyltransferase protein KAT6A were respectively detected in a NCC and a PCC of this protein. Both mutations were shown to be involved in mental retardation and intellectual disability syndrome by causing a truncation within the acidic domain of the KAT6A protein. The mutant *KAT6A* allele alters global acetylation of histones H3K9 and H3K18 and affects P53-mediated pathways in apoptosis, metabolism, and transcriptional regulation [33].

Remarkably, variants occurring in positively charged regions are more often pathogenic than those occurring in negatively charged ones. Which leads us to conclude that the presence of variants in PCCs has generally a negative impact compared to NCCs. We suggest that this is due to the fact that PCCs may include Nuclear Localisation Sequences (NLSs), therefore a mutation in these sequences could prevent the addressing of the protein to the nucleus. It is worth to say that the NCBI data used in this study were not exhaustive and that further investigations are needed to confirm this hypothesis and to improve our knowledge on mutation effects on proteins and pathogenicity.

Interestingly, we found that clusters of both charges are variant rich regions since on average we obtained a variant every 4 or 5 bp in NCC and PCC respectively. Besides, the count of variants occurring in the encoding regions of CCs is on average bigger than that found in their corresponding genes and full mRNAs. This is consistent with the results reported by Wooton [34] who found that low-complexity regions, such as charged regions, are subject of rapid evolution by molecular processes such as recombinational repeat expansion, deletion, replication slippage and a high frequency of substitution mutations.

Furthermore, according to de Beer et al. [35], the variants occur predominately on the surface of protein (82%) explaining further the richness of CCs in variants content, since charged regions in protein are mainly exposed at the surface playing a role at the interfaces with other proteins or nucleic acids [36, 37]. Moreover, our results make it clear that the NCCs are significantly richer in variants compared to PCCs (*p* = 0,003) even when adjusting for the length of CC (note that NCCs are on average significantly longer than PCCs) [3].

In general, charged amino acids mutability is an ambiguous issue. While Dayhoff et al. [28] and Jones et al. [31] consider that charged amino acids, particularly acidic ones, are highly mutable, Majewski and Ott [38] showed that these amino-acids appear to be relatively non-mutable. In our study, we found that the acidic and basic amino acids are the most mutated (Fig. 5), which is obvious since the studied charged regions are biased in favour of charged amino acids. In particular, the acidic and basic residues tend to be substituted by residues sharing the same physicochemical properties (polar side chain). For example, the substitutions within negatively charged regions tend strongly to increase polarity (2999 polar) against hydrophobicity (388 hydrophobic). These findings reflect a selection imposed by the fact that more extreme differences in hydrophobicity are disease-associated variants [35].

The substitution frequency of negative residues by positive ones is significantly similar to that of substitution of negative residues by negative ones (~0.24 versus ~ 0.23). Likewise, the substitution frequency of positive residues by positive ones is significantly similar to that of substitution by negative ones (~0.09 versus ~ 0.06). However, it is easier for a negative residue to be substituted by a positive one (frequency = 0.237) than the inverse (a positive substituted by a negative, frequency = 0.058). Conversely, Majewski and Ott [38] found that substitutions generally do occur more easily within groups (hydrophobic, polar, basic; acid) than between groups and that the least permissive type of substitutions between groups is basic to acidic occurring at 35% of the neutral rate. By comparing our results to those of Majewski and Ott [38] we deduce that these charged regions have widely different features.

All exchanges between basic and acidic residues are substitutions between Glu and Lys. This may be due to the fact that it is easy to substitute any base of the two codons of the Glu (GAA, GAG) to obtain those of Lys (AAA, AAG) and vice versa. However, 74% of substitutions of basic residues by hydrophobic occurred between Arg and Trp. This may be also explained by the similarity between Arg codons mostly AGG and CGG, and the unique codon TGG of Trp. Despite the fact that this type of exchanges is the most widespread among substitutions between positive and hydrophobic, but does not prevent their deleteriousness [38] along with substitutions from Arg to Cys which represent 30% of substitutions of positive by polar residues.

**Fig. 6:**
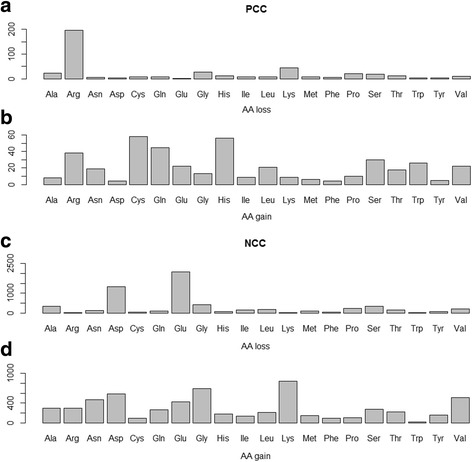
Pattern of aa loss (**a**, **c**) and gain (**b**, **d**) in PCCs and NCCs respectively. The plots show the difference between how often an amino acid is substituted versus how often it is substituted to. The bars show the number of occurrence of substitution of each amino acid. aa: amino-acid, PCCs: Positive charge clusters, NCCs: Negative charge clusters. The plot shows that the majority of variants are rare (MAF < 0.005; *p* = 5 × 10^–^^5^)

Within PCC, the basic residues are the greatest loser group whereas polar residues are the greatest gainers (Fig. 6a and b). Correspondingly, within NCC, the acidic and the polar residues are respectively the biggest losers and gainers (Fig. 6c and d). The pattern of amino acid gain and loss in CCs shows that in basic regions, Arg is the greatest loser (Fig. 6a) and the Cys is the greatest gainer (Fig. 6b), while in NCCs, Glu and Lys are respectively the greatest loser and gainer (Fig. 6c and d). Our results confirm previous findings by Zuckerkandl et al. [39] who reported that Cys is being accrued significantly in human proteome and Arg is the strongest loser. In the same study, Lys was identified as a weak loser or a gainer but our NCC dataset suggests that it is the strongest gainer among all residues.

Insertions also occur to the benefit of the charged residues in both positive and negative CCs; actually there were no inserted hydrophobic residues in the set of PCCs, suggesting that insertion occurs according to the amino-acid environment. Of course, the occurrence of insertions was found to decrease according to the number of inserted residues which makes the one-amino-acid insertions the most frequent. This is expected since natural selection tends to minimize the count of inserted bases. Actually, as well as constraints on the mutational process at the DNA level, the consequence of a variant on the protein structure and function will also have an impact on the number of observed mutations.

Despite the fact that the charged residues are the most inserted, they are the most deleted ones as well. We think that is quite natural because they are the most prevalent. It is worth noting that deletions are much more frequent than insertions (e.g. deletions are three times more prevalent than insertions within NCCs). We know that in cancer-associated mutations, deletions occur more than insertions [40]. Consequently, it is strongly needed to carry out a more in-depth studies in order to understand the mechanism of deletion, as well as the possible consequences of these mutations and their involvement in different types of cancer and other diseases.

Finally, charged amino-acids are also the most affected by synonymous variants which are in general neutral mutations, although replacing an efficient codon with a less efficient synonymous one can affect translation rate and consequently the amount of the protein.

## Conclusions

The results presented herein emphasize on genetic variation within charged regions in the human proteome at the DNA and protein levels which is considered to be the first study of its kind.

Human charge clusters were found to be conserved since their variants were mainly rare compared to their background sequences (gene and protein), which suggests that these regions are under high selection pressure. However, further investigations and experimental studies have to be carried out in order to understand the possible impact of these sequence variations on the translation, structure and function of the protein.

Moreover, our findings provide insights into the distinctive features of CCs compared to the whole proteome in regards to the occurrence of mutations and SNPs, namely substitution and Indels; this supports the interest that must be given for a better understanding of those charged regions.

## Additional files

Additional file 1: Occurrence of charge clusters in the human proteome. The charge clusters were detected by the FCCP program [3]. (PDF 11 kb)

Additional file 2: Scripts used in the study. (PDF 12 kb)

Additional file 3: The steps of the workflow allowing to collect the charge cluster variants datasets. All steps, databases and bioinformatics tools used are described and detailed in the Materials and Methods section. (TIFF 6375 kb)

Additional file 4: Distribution of NCCs within genes. (PNG 63 kb)

Additional file 5: Distribution of PCCs within genes. (PNG 64 kb)

Additional file 6: Insertions occurring within negative charge clusters (NCCs). (XLSX 10 kb)

Additional file 7: Pie showing the occurrence of synonymous variation according to residues within negative charged clusters. The plot shows that Glutamic and aspartic acids are the most affected residues by this type of variation. (TIFF 6374 kb)

Additional file 8: Pie showing the occurrence of synonymous variation according to residues within positive charged clusters. The plot shows that Arginine (32%) is by far the most affected residue by this type of variation and that Proline is the second most affected one. (TIFF 6374 kb)

## Abbreviations

Aa: Amino acid
Ala: Alanine
ANOVA: ANalysis Of VAriance
Arg: Arginine
Asn: Asparagine
Asp: Aspartic acids
Bp: base pair
CC: Charge Cluster
Cys: Cysteine
dbSNP: SNP database
dbVar: Genomic structural variation database
DNA: Deoxyribonucleic acid
Ex-AC: Exome Aggregation Consortium
FCCP: Finding Charge Clusters in Protein sequences
Gln: Glutamine
Glu: Glutamic acid
Gly: Glycine
GO-ESP: Grand Opportunity Exome Sequencing Project
His: Histidine
HSD: Honest Significant Difference
Ile: Isoleucine
Leu: Leucine
Lys: Lysine
MAF: Minor Allele Frequency
Met: Methionine
mRNA: messenger ribonucleic acid
NCC: Negative Charge Cluster
NLS: Nuclear Localization Sequence
*P*: *P*-value
PCC: Positive Charge Cluster
Phe: Phenylalanine
Pro: Proline
RNA: Ribonucleic acid
Ser: Serine
SNP: Single Nucleotide Polymorphism
SNV: Single Nucleotide Variation
Thr: Threnonine
Trp: Tryptophan
Tyr: Tyrosine
Val: Valine
VV: Variation Viewer. Amino acid three letter codes

## References

[1] Karlin S, Blaisdell BE, Brendel V (1990). Identification of significant sequence patterns in proteins. Methods Enzymol.

[2] Karlin S (1995). Statistical significance of sequence patterns in proteins. Curr Opin Struct Biol.

[3] Belmabrouk S, Kharrat N, Benmarzoug R, Rebai A (2015). Exploring proteome-wide occurrence of clusters of charged residues in eukaryotes. Proteins.

[4] Aifa S, Miled N, Frikha F, Aniba MR, Svensson PSS, Rebai A (2006). Electrostatic interactions of peptides flanking the tyrosine Kinase domain in the epidermal growth factor receptor provides a model for intracellular dimerization and autophosphorylation. Proteins.

[5] Choura M, Rebaï A (2017). Exploring disorder in the human charged biased proteins. J Recept Sig Transd.

[6] Karlin S (2005). Statistical signals in bioinformatics. Proc. Natl. Acad. Sci. U S A..

[7] Sheinerman FB, Norel R, Honig B (2000). Electrostatic aspects of protein–protein interactions. Curr Opin Struct Biol.

[8] Torshin IY, Harrison RW (2001). Charge centers and formation of the protein folding core. Proteins.

[9] Hurst JM, McMillan LE, Porter CT, Allen J, Fakorede A, Martin AC (2009). The SAAPdb web resource. A large scale structural analysis of mutant proteins. Hum. Mutat.

[10] Vreken PV, Van Kuilenburg ABP, Meinsma R, Van Gennip AH (1997). Dihydropyrimidine dehydrogenase (DPD) deficiency. Identification and expression of missense mutations C29R, R886H and R235W. Hum. Genet.

[11] Teng S, Madej T, Panchenko A, Alexov E (2009). Modeling effects of human single nucleotide polymorphisms on protein-protein interactions. BiophysJ.

[12] Sunyaev S, Ramensky V, Koch I, Lathe W, Kondrashov AS, Bork P (2001). Prediction of deleterious human alleles. Hum Mol Gen.

[13] Shirley BA, Stanssens P, Hahn U, Pace CN (1992). Contribution of hydrogen bonding to the conformational stability of ribonuclease T1. Biochemistry.

[14] Wang Z, Moult J (2001). SNPs, protein structure, and disease. Hum Mutat.

[15] Uniprot: http://www.uniprot.org/ release 2016_02.

[16] BLAST https://blast.ncbi.nlm.nih.gov/Blast.cgi

[17] GenBank https://www.ncbi.nlm.nih.gov/gene release 212.

[18] RefSeq https://www.ncbi.nlm.nih.gov/refseq/ release 74.

[19] Variation Viewer https://www.ncbi.nlm.nih.gov/variation/view/ release 1.5.

[20] Brendel V, Bucher P, Nourbakhsh IR, Blaisdell BE, Karlin S (1992). Methods and algorithms for statistical analysis of protein sequences. Proc. Natl. Acad. Sci. U S A.

[21] Choura M, Rebaï A (2013). Exploring charged biased regions in the human proteome. Gene.

[22] Brendel V, Karlin S (1989). Association of charge clusters with functional domains of cellular transcription factors. Proc Natl Acad Sci U S A.

[23] Alba MM, Santibánez-Koref MF, Hancock JM (1999). Amino acid reiterations in yeast are overrepresented in particular classes of proteins and show evidence of a slippage-like mutational process. J Mol Evol.

[24] Karlin S, Brocchieri L, Bergman A, Mrázek J, Gentles AJ (2002). Amino acid runs in eukaryotic proteomes and disease associations. Proc Natl Acad Sci U S A.

[25] Karlin S, Burge C (1996). Trinucleotide repeats and long homopeptides in genes and proteins associated with nervous system disease and development. Proc Natl Acad Sci U S A.

[26] Tennessen JA, Bigham AW, O’Connor TD, Fu W, Kenny EE, Gravel S, McGee S, Do R, Liu X, Jun G, Kang HM, Jordan D, Leal SM, Gabriel S, Rieder MJ, Abecasis G, Altshuler D, Nickerson DA, Boerwinkle E, Sunyaev S, Bustamante CD, Bamshad MJ, Akey JM, Broad GO, Seattle GO (2012). Evolution and functional impact of rare coding variation from deep sequencing of human exomes. Science.

[27] McLachlan AD (1972). Repeating sequences and gene duplication in proteins. J Mol Biol.

[28] Dayhoff MO, Schwartz RM, Orcutt BC, Dayhoff MO (1978). A model of evolutionary change in proteins. atlas of protein sequence and structure.

[29] Henikoff S, Henikoff JG (1992). Amino acid substitution matrices from protein blocks. Proc Natl Acad Sci U S A.

[30] Johnson MS, Overington JP. A structural basis for sequence comparisons:an evaluation of scoring methodologies. J Mol Biol. 1993;233:716–38. 10.1006/jmbi.1993.1548 8411177

[31] Jones DT, Taylor WR, Thornton JM (1994). A mutation data matrix for transmembrane proteins. FEBS Lett.

[32] Tham E, Lindstrand A, Santani A, Malmgren H, Nesbitt A, Dubbs HA, Wilson GN (2015). Dominant mutations in KAT6A cause intellectual disability with recognizable syndromic features. Am J Hum Genet.

[33] Arboleda VA, Lee H, Dorrani N, Zadeh N, Willis M, Macmurdo CF, Miceli MC (2015). De novo nonsense mutations in KAT6A, a lysine acetyl-transferase gene, cause a syndrome including microcephaly and global developmental delay. Am J Hum Genet.

[34] Wootton JC (1994). Sequences with ‘unusual’ amino acid compositions. Curr Opin Struct Biol.

[35] de Beer TA, Laskowski RA, Parks SL, Sipos B, Goldman N, Thornton JM (2013). Amino acid changes in disease-associated variants differ radically from variants observed in the 1000 genomes project dataset. PLoS Comput Biol.

[36] Fujita H, Yamagishi M, Kida Y, Sakaguchi M (2011). Positive charges on the translocating polypeptide chain arrest movement through the translocon. J Cell Sci.

[37] Karlin S, Zhu ZY (1996). Characterizations of diverse residue clusters in protein three-dimensional structures. Proc Natl Acad Sci U S A.

[38] Majewski J, Ott J (2003). Amino acid substitutions in the human genome: evolutionary implications of single nucleotide polymorphisms. Gene.

[39] Zuckerkandl E, Derancourt J, Vogel H (1971). Mutational trends and random processes in the evolution of informational macromolecules. J Mol Biol.

[40] Iengar P (2012). An analysis of substitution, deletion and insertion mutations in cancer genes. Nucleic Acids Res.

