# Supplementary material for: Screening of nucleotide variations in genomic sequences encoding charged protein regions in the human genome
Source: BMC Genomics. 2017 Aug 8;18:588. doi: 10.1186/s12864-017-4000-3 (PMC5549384; doi:10.1186/s12864-017-4000-3)
Supplement: Supplementary file 1 — Occurrence of charge clusters in the human proteome. The charge clusters were detected by the FCCP program [3]. (PDF 11 kb) [file 12864_2017_4000_MOESM1_ESM.pdf]

**Table 1.** Occurrence of charge clusters in the human proteome. The charge clusters were detected by the FCCP program (Belmabrouk et al., 2015).

| | **Positive charge clusters** | **Negative charge clusters** |
|---|---|---|
| **Total count of charge clusters** | 526 | 1628 |
| **Protein count*** | 498 | 1435 |
| **Protein average size¥** | 781.3 ± 732.6 | 883.4 ± 1095.67 |
| **Cluster average size** | 24.23 ± 3.9 | 27.8 ± 7.6 |
| **Average count of CC per protein** | 1 | 1 |

*Sequences having NCCs and PCCs
¥average size : mean ± standard deviation
NCC: Negative Charge Cluster
PCC: Positive Charge Cluster
