# Supplementary material for: Screening of nucleotide variations in genomic sequences encoding charged protein regions in the human genome
Source: BMC Genomics. 2017 Aug 8;18:588. doi: 10.1186/s12864-017-4000-3 (PMC5549384; doi:10.1186/s12864-017-4000-3)
Supplement: Supplementary file 2 — Scripts used in the study. (PDF 12 kb) [file 12864_2017_4000_MOESM2_ESM.pdf]

**Table S1.** *Scripts used in the study.*

| R Scripts | Input | Output |
|---|---|---|
| **Script 1** | • Charge cluster dataset obtained by FCCP program (Belmabrouk *et al.*, 2015)<br>• «Gene table» (NCBI / Gene) | List of CC and protein coordinates on the gene |
| **Script 2** | • Script (1) output<br>• Variation Viewer | List of SNPs by Gene |
| **Script 3** | • CC lists (Script (1) output)<br>• SNP lists (script (2) output) | For each CC, it provides data frames containing SNP data:<br>• Molecular consequence<br>• Clinical significance<br>• Variant types<br>• 1000 Genomes MAF<br>• Ex-AC MAF<br>• GO-ESP MAF |
| **Script 4** | • NCC list (Script (3) output)<br>• PCC list (Script (3) output) | List of CCs in proteins having both types of charge clusters. |
| **Script 5** | • Variant list | Variant classified according variant types and amino acid groups |

CC: Charge Cluster
FCCP: Find Charge Clusters in Protein sequences
SNP: Single Nucleotide Polymorphism
MAF: Minor Allele Frequency
