# Supplementary figures and images for: Screening of nucleotide variations in genomic sequences encoding charged protein regions in the human genome
Source: BMC Genomics. 2017 Aug 8;18:588. doi: 10.1186/s12864-017-4000-3 (PMC5549384; doi:10.1186/s12864-017-4000-3)

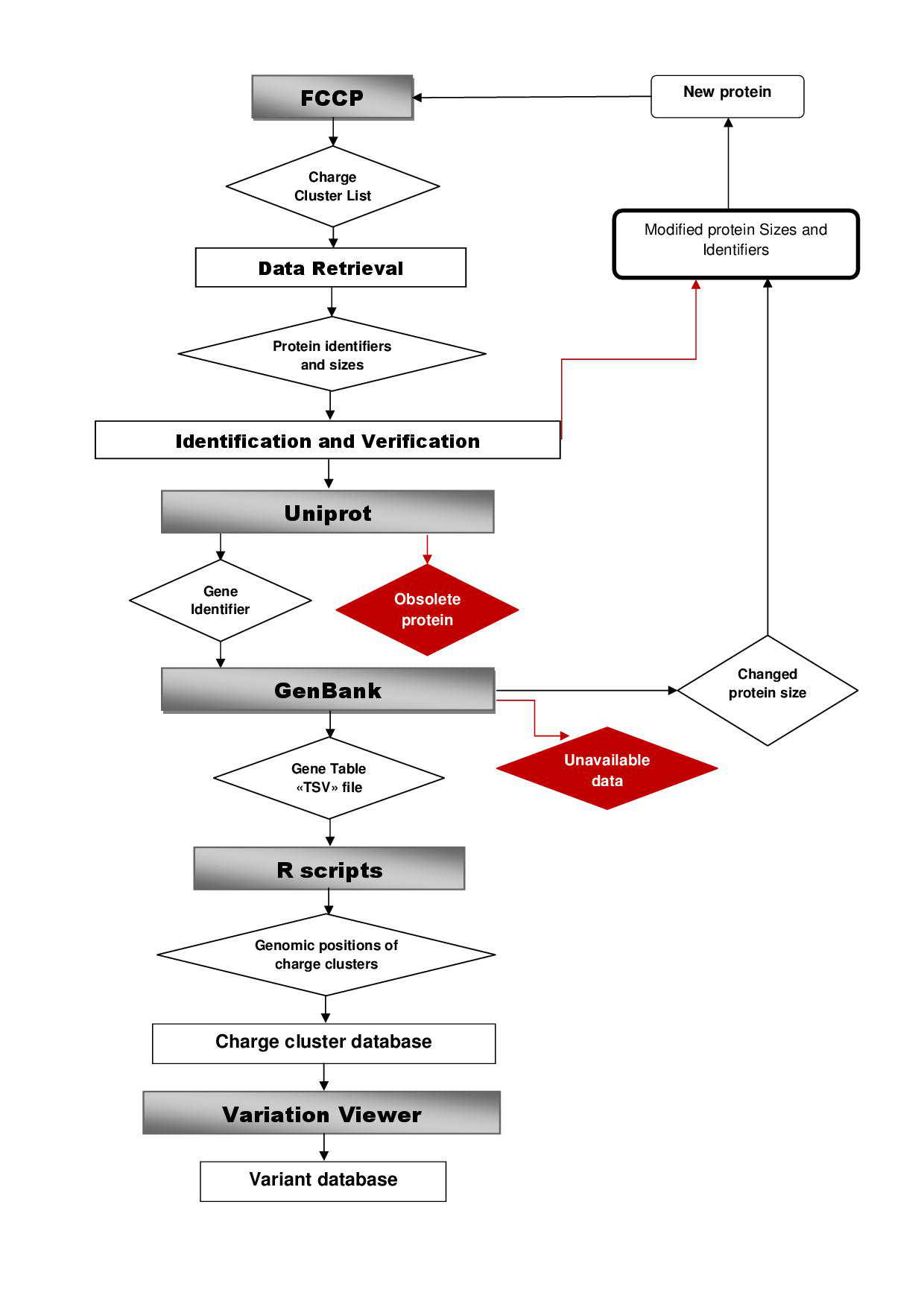

Supplement: Supplementary file 3 — The steps of the workflow allowing to collect the charge cluster variants datasets. All steps, databases and bioinformatics tools used are described and detailed in the Materials and Methods section. (TIFF 6375 kb) [file 12864_2017_4000_MOESM3_ESM.tif]

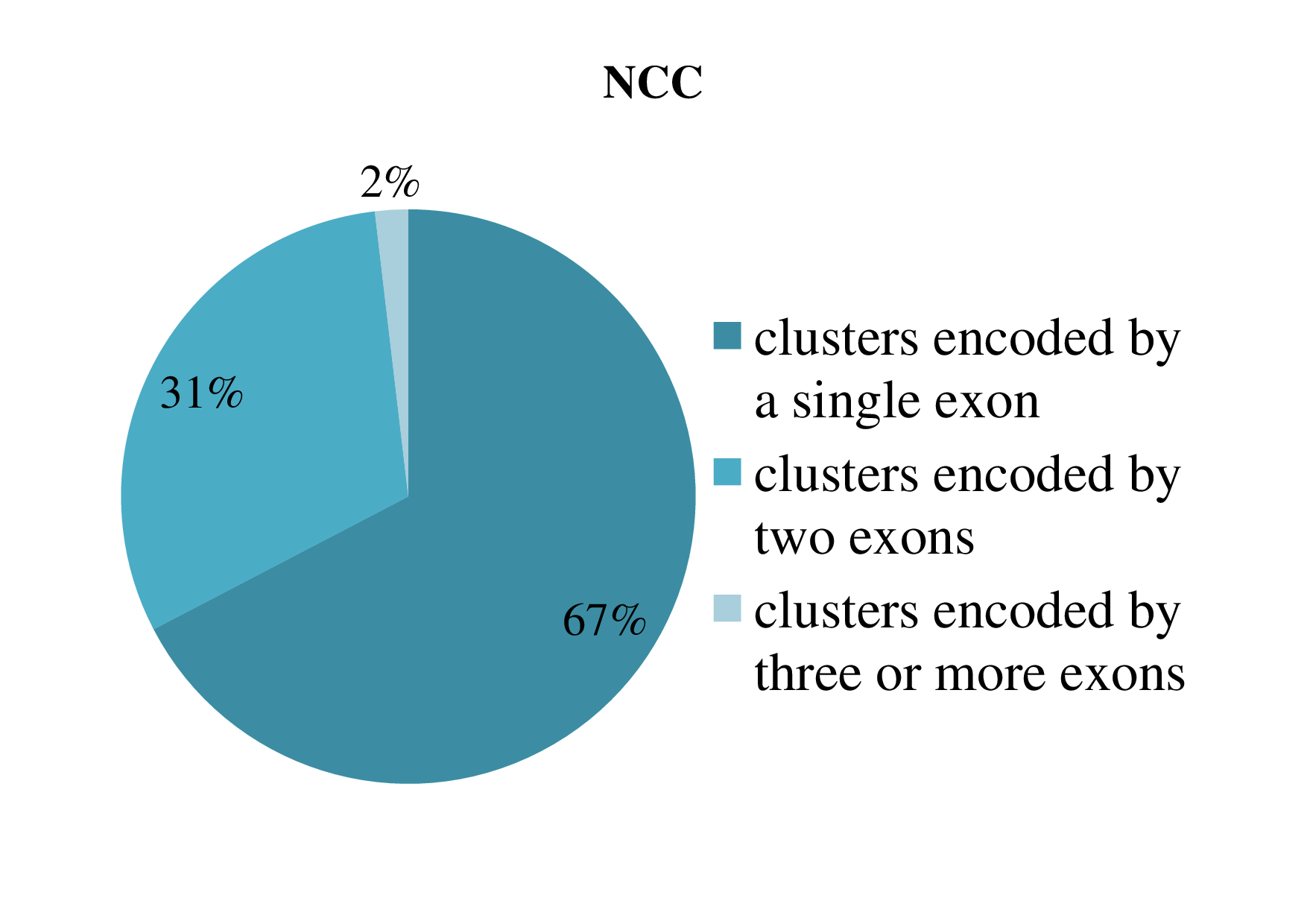

Supplement: Supplementary file 4 — Distribution of NCCs within genes. (PNG 63 kb) [file 12864_2017_4000_MOESM4_ESM.png]

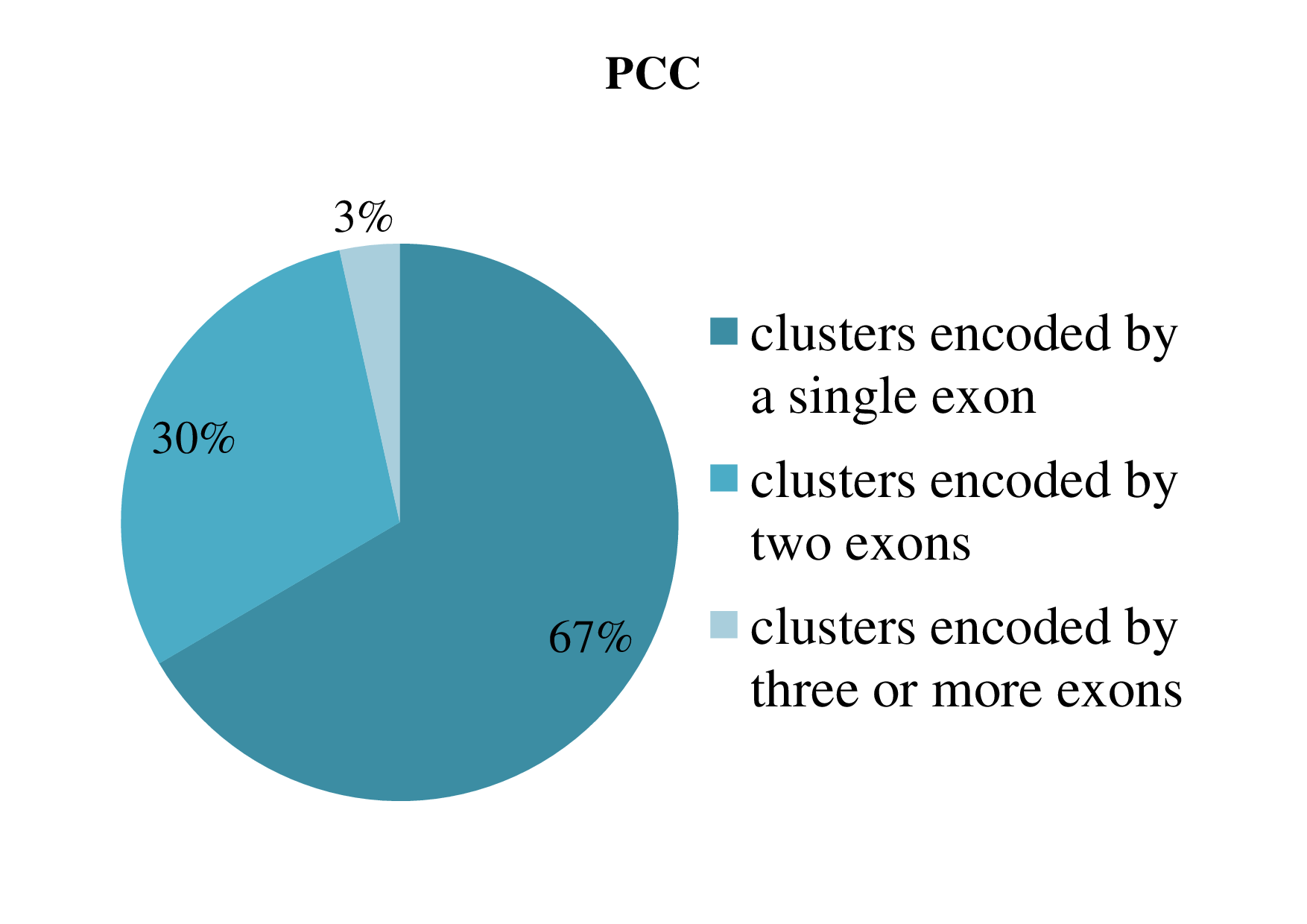

Supplement: Supplementary file 5 — Distribution of PCCs within genes. (PNG 64 kb) [file 12864_2017_4000_MOESM5_ESM.png]

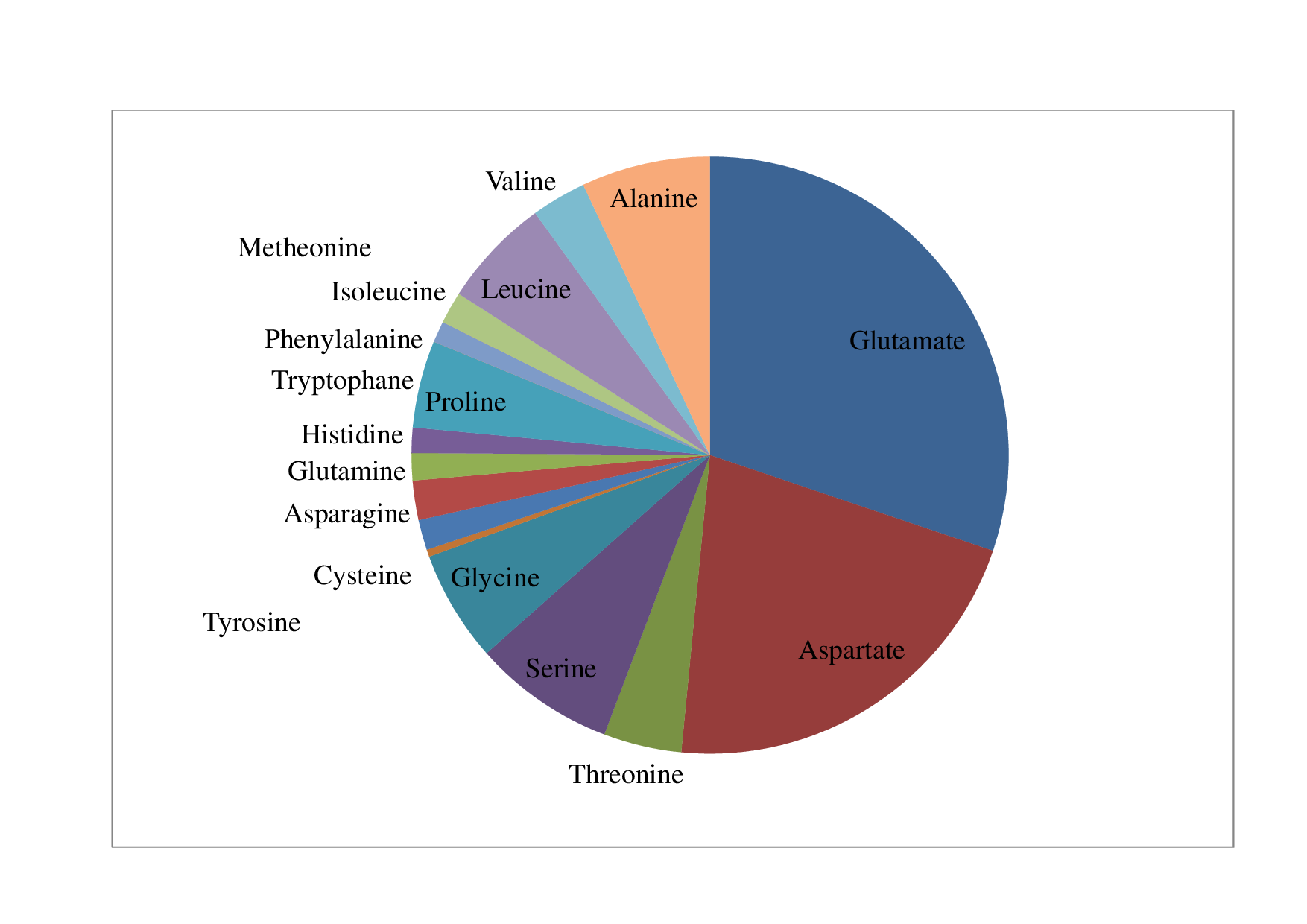

Supplement: Supplementary file 7 — Pie showing the occurrence of synonymous variation according to residues within negative charged clusters. The plot shows that Glutamic and aspartic acids are the most affected residues by this type of variation. (TIFF 6374 kb) [file 12864_2017_4000_MOESM7_ESM.tif]

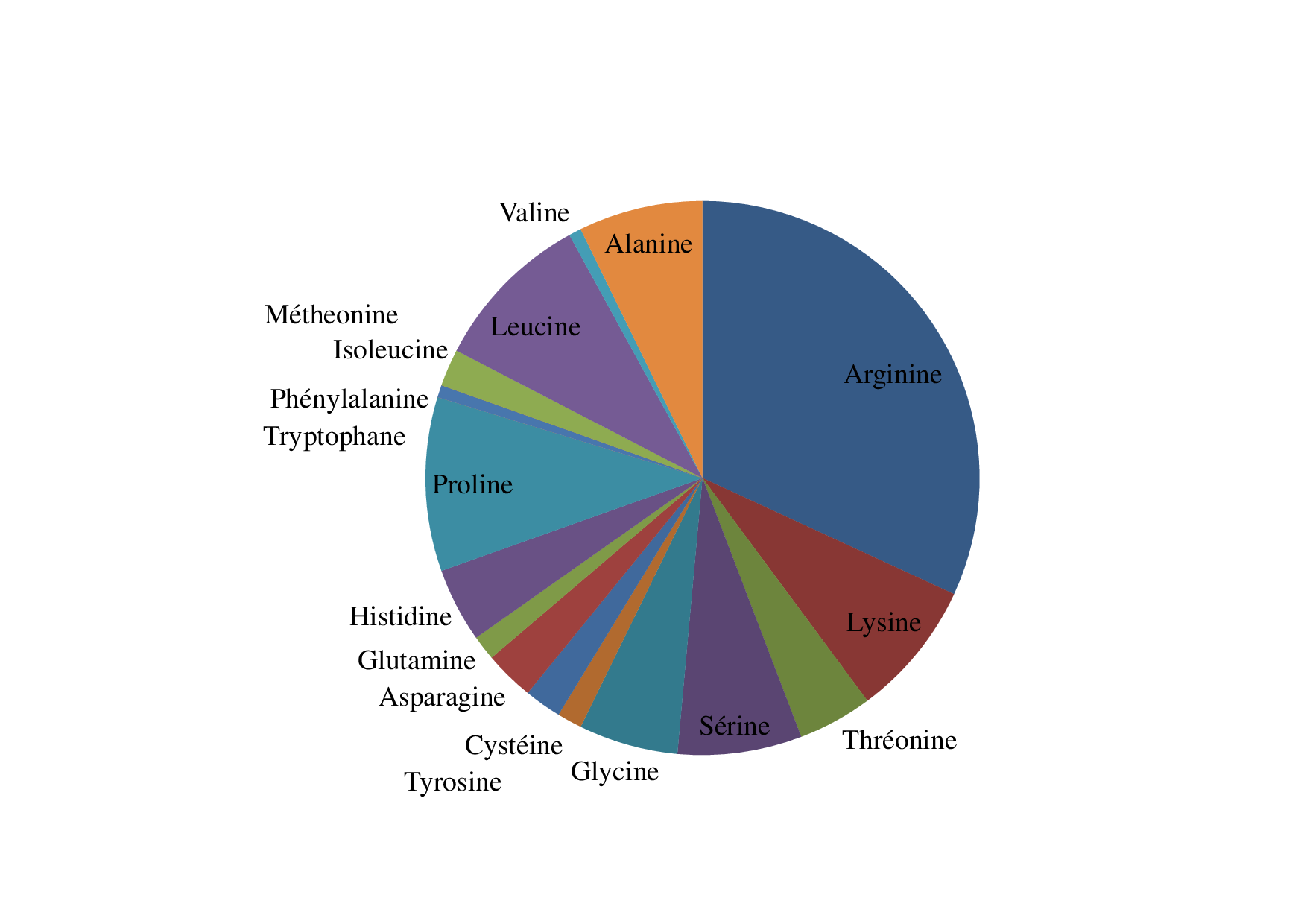

Supplement: Supplementary file 8 — Pie showing the occurrence of synonymous variation according to residues within positive charged clusters. The plot shows that Arginine (32%) is by far the most affected residue by this type of variation and that Proline is the second most affected one. (TIFF 6374 kb) [file 12864_2017_4000_MOESM8_ESM.tif]
